# A Case Series on Critically Ill Pregnant or Newly Delivered Patients with Covid-19, Treated at Karolinska University Hospital, Stockholm

**DOI:** 10.1155/2021/8868822

**Published:** 2021-02-10

**Authors:** Rasha El-ahmad Polcer, Elin Jones, Karin Pettersson

**Affiliations:** Department of Obstetrics and Gynecology, Karolinska University Hospital, Stockholm, Sweden

## Abstract

In this retrospective report, we present five cases of critically ill pregnant or newly delivered women positive for Covid-19 admitted to our obstetrical departments at Karolinska University Hospital. They compose 6% of eighty-three pregnant women that tested positive for SARS-CoV-2 during the period March 25 to May 4, 2020. Three patients were at the time of admission in gestational week between 21 + 4 and 22 + 5 and treated during their antenatal period; meanwhile, the other two were admitted within 1 week postpartum. All of them were in need of intensive care: one was treated with high flow oxygen therapy, the other four with invasive mechanical ventilation (three with endotracheal intubation and one with extracorporeal membrane oxygenation). Age above thirty, overweight, and gestational diabetes are notable factors in the cases presented. At the time of admission, they all presented with symptoms such as fever, cough, and dyspnea. Chest imaging with computer tomography scan was performed in each case and demonstrated multifocal pneumonic infiltrates in all of them, but no pulmonary embolism was confirmed in any. Neither did the echocardiogram indicate any cardiomyopathy. Four of the patients have been discharged from the hospital, with an average of 20 hospital days. One antenatal pregnant woman needed prolonged ECMO therapy; in gestational week 27 + 3, she went into cardiac arrest, resulting in an urgent C-section on maternal indication. At the time of writing, she is still hospitalized. In coherence with other published reports, our cases indicate that critically ill pregnant women infected by SARS-Cov-2 may develop severe respiratory distress syndrome requiring prolonged intensive care. The material is limited for conclusions to be made; more detailed information on symptoms, treatment, and outcomes for pregnant and postpartum women managed in intensive care is therefore needed.

## 1. Introduction

It is by now known that the pneumonia of unknown cause that was detected in Wuhan, China, reported to the World Health Organization (WHO) County Office on December 31, 2019, was caused by a new coronavirus strain, SARS-CoV-2. The outbreak was declared as a Public Health Emergency of International Concern on January 30, 2020, and the new coronavirus disease was named Covid-19 [1]. Today, the virus has spread all over the world, and the exact number of infected individuals is not known. Globally, more than 25 million people have been confirmed infected with SARS-CoV-2, and more than 850 thousand people have died from Covid-19 [2].

Risk factors for developing symptomatic Covid-19 and severe respiratory distress syndrome include age above 70 years and, in addition, a medical condition such as diabetes, chronic respiratory disease, cardiovascular and kidney disease, impaired immune system, obesity, and neuromuscular disease [3].

Initially, it was not clearly known if pregnant women were at increased risk of developing severe symptoms from Covid-19 compared to the nonpregnant female population, as little data and information had been published. Initial studies concluded that pregnant women shared the same clinical characteristics as nonpregnant women and there was not enough evidence to support that pregnant women had an increased risk of developing severe disease compared to the nonpregnant women in the same age groups [4, 5].

As more data has been collected, several reports and case series indicate that pregnant women may be at higher risk of becoming critically ill and requiring higher intensive care. Therefore, careful monitoring of pregnant women with Covid-19 has been warranted [6]. There have also been reports of maternal deaths due to Covid-19 [7].

In May 9, 2020, the Swedish Public Health Agency published a report on pregnant and early postpartum (<1 week postpartum) women diagnosed with Covid-19 who required intensive care during the period between March 19 and April 20, 2020. The relative risk for pregnant and postpartum women compared to nonpregnant women was based on the estimated number of pregnant and nonpregnant women in the Swedish population. A total of fifty-three women, nonpregnant and pregnant, between the age of 20 and 45 years, received intensive care according to the Swedish Intensive Care Registry. Thirteen of them were or had recently been pregnant. Although the result was based on a relatively small number of cases, the estimated risk for pregnant women to receive intensive care was significantly higher than the nonpregnant population (14.4 per 100,000 (95% CI 7.3-23.4) for pregnant/postpartum women and 2.5 per 100,000 (95% CI 1.8-3.5) for nonpregnant women in the same age group). As a result, pregnant women were recommended to take necessary precautions to avoid infection during pregnancy [8, 9].

The region most affected by Covid-19 in absolute numbers in Sweden is Stockholm [10]. The Karolinska University Hospital has been the main center for admitting pregnant women and recently delivered women, who required intensive care. Since March 25, 2020, of all the women admitted to the wards at the two obstetrical units, Karolinska Huddinge and Karolinska Solna, 2 have been screened for Covid-19 with a polymerase chain reaction (PCR) test. This was implemented as a safety routine for the protection of the medical staff working near the patients during labor as well as during urgent surgical procedures.

During the period between March 25 and May 4, 2020, 885 delivered pregnant women were tested for Covid-19. 7.2%, 64, tested positive; out of these, 76.6%, 49, were asymptomatic and 23.4%, 15, were symptomatic with either mild, moderate, or severe symptoms. Eleven symptomatic patients have been in need of extended hospital care due to moderate or severe symptoms related to Covid-19 disease. Eight had moderate symptoms and were observed on regular medical wards. Three were admitted to the intensive care and in need of invasive mechanical ventilation support. In addition, two antenatal pregnant women who tested positive during the same period needed intensive care due to Covid-19.

Since there is a worldwide interest and need of a better understanding on how SARS-CoV-2 affects pregnant women, especially in those that get critically ill, we decided to present a case series on five pregnant women who needed intensive care due to severe or critical Covid-19.

## 2. Methods

### 2.1. Study Design and Participants

This is a retrospective case series of symptomatic pregnant women that tested positive for SARS-CoV-2 with quantitative reverse transcription polymerase chain reaction (qRT-PCR) during the period March 25 and May 4, 2020. Fifteen symptomatic pregnant women required hospital care during the period, at Karolinska University Hospital, Stockholm, Sweden. In this report, we decided to include those pregnant women who at least had entered their second trimester at the time of admission to the hospital or were <1 week postpartum and who developed severe or critical Covid-19 requiring intensive care, resulting in five cases.

The definition of severe and critical disease is according to the report of the WHO-China Joint Mission on Coronavirus Disease 2019. Severe disease is described as dyspnea, respiratory frequency ≥ 30 breaths/min, blood oxygen saturation ≤ 93% or alveolar oxygen partial pressure (PaO_2_)/fraction of inspiration oxygen (FIO_2_) ≤ 300 mmHg, and/or lung infiltrates > 50% of the lung field within 24-48 hours. Critical disease is described as respiratory failure, septic shock, and/or multiple organ dysfunction/failure [11].

### 2.2. Case 1

A 29-year-old, gravida 1, para 0, with Middle Eastern background, at week 21 + 4 of gestation presented at the emergency ward with dry cough and upper respiratory tract symptoms lasting for more than three months. She had sought help at an outpatient clinic two times previously. The previous seven days, the patient developed fever, nausea, and diarrhea and felt an augmentation in her cough. She had no previous medical history; her only medication was prenatal vitamins and iron. Her body mass index (BMI) was 25.6 kg/m^2^.

At the time of admission to the hospital, she was fatigued, had a fever of 39.5°C, oxygen saturation (SpO_2_) of 94%, respiratory rate (RR) of 24 breaths/minute, and heart rate (HR) 118 beats/minute, and her blood pressure (BP) was 110/78 mmHg. She tested positive for Covid-19 with a PCR test. During the first four hospital days (HD), she did not require any specific medical support or medication besides cough medication. Prophylactic antithrombotic treatment with low molecular- weight-heparin (LMWH) was initiated. Her fever decreased, and the cough got better, but at the fifth HD, the cough got worse, she developed epigastric pain, nausea, and dyspnea and required increased oxygen levels. Her c-reactive protein (CRP) and procalcitonin (PCT) levels increased, and treatment with intravenous antibiotics was induced due to suspected bacterial pneumonia.

At HD 8, the patient*'*s oxygen demand increased to 10 L O_2_ on an oxymask to maintain saturation > 92%, her respiratory rate was 32-40 beats/min, and her arterial blood gas (ABG) indicated moderate acute respiratory distress syndrome (ARDS) [12]. She was transported to the intensive care unit (ICU) and put on nasal high flow therapy (NHF) 60%, O_2_ 12 L/min, with endotracheal intubation as a backup. The LMWH dose was doubled as a routine in critically ill pregnant women to prevent thrombotic event. Her potassium level decreased to 2.8 mmol/L, and her aminotransferase levels increased. She was given potassium infusion, in addition to intravenous fluids and parenteral nutrition. Daily fetal heart rate was monitored and normal; no prednisolone or other obstetrical intervention was therefore made at that time. Chest imaging, with CT scan, demonstrated multifocal pneumonic infiltrates, but no pulmonary edema or embolism.

The patient became increasingly better; no endotracheal intubation was needed. On HD 11, she was transferred to a medical ward, and on HD 15, gestational week 23 + 6, she was discharged from the hospital. The patient is today in gestational week 28 + 1 with maintained fatigue but no cough. The latest ultrasonographic fetal biometry in gestational week 26 + 3 indicated normal fetal growth, and there were no signs of fetal hypoxia on the Doppler examination.

### 2.3. Case 2

A 36-year-old, gravida 5, para 2, with African background, and previous normal deliveries, expecting dichorionic/diamniotic duplex (DC/DA) presented at gestational week 22 + 5 at the obstetrical emergency ward with cough, fatigue, arthralgia, myalgia, nausea, and vomiting. The symptoms had first occurred eight days earlier, but now she had worsened; she had not been able to maintain any nutrition and appeared dehydrated. She had developed gestational hypothyroidism and gestational diabetes in this pregnancy. Her BMI was 29.69 kg/m^2^. Her medication included levothyroxine, insulin, iron, and prenatal vitamins.

At the time of admission to the hospital, she had, in addition to the symptoms previously mentioned, tachycardia (HR 130 beats/minute), hypovolemia (BP 94/61 mmHg), and tachypnea (RR 28 breaths/minute) but no hypoxemia (SaO_2_ 98%) and no fever. The ABG indicated acute starvation ketoacidosis and hypokalemia (potassium 2.8 mmol/L); the CRP was elevated, and in addition, she also had proteinuria, ketonuria, leukocyturia, and glucosuria. No other organ dysfunction or signs of preeclampsia was seen on the blood samples. She was transported to the intermediate medical care (IMC: step-down unit from highly intensive care) unit for fluid resuscitation, and prophylactic treatment with LMWH was initiated. She tested positive for Covid-19 with PCR.

Over the next 3 days, she begun to deteriorate in her respiration with dyspnea and increased RR, and at least 2 L O_2_ on nasal cannula was needed to maintain an SaO_2_ above 95%. On HD 5, her oxygen demand increased to at least 5-6 L of O_2_ on mask to maintain a SaO_2_ above 95%. Chest imaging with CT scan demonstrated multifocal pneumonic infiltrates affecting 50% of the right lung and 30% of the left lung, but no pulmonary embolism was seen. The echocardiogram demonstrated hyperdynamic left ventricle indicating hypovolemia. She was transferred to the ICU, and endotracheal intubation was performed due to moderate ARDS. The first dose of antenatal betamethasone for fetal lung maturation was given, and prophylactic LMVH was doubled. Intravenous antibiotics were started due to high PCT levels indicating bacterial super infection.

During the time she was treated at the ICU, her clinical status initially worsened with increased oxygen demand and ventilation support. Fetal monitoring was limited to heart rate auscultation once per day, and she was allowed to lie in the prone position to improve the ventilation.

She gradually recovered, and on HD 16 (ICU day 12), a successful extubation was achieved. On HD 17, step-down from ICU to IMC was performed, and she was transferred to a medical ward for rehabilitation. At HD 22, in gestational week 25 + 5, she was discharged from the hospital.

At the moment of writing, the patient has fully recovered from the respiratory insufficiency. She went through several ultrasonographic fetal biometrics which indicated normal growth in both twins, and in gestational week 38 + 1, she was delivered with a planned cesarean section.

### 2.4. Case 3

A 30-year-old, gravida 2, para 1, with Asian background presented at the obstetrical ward in gestational week 37 + 4 with spontaneous start of labor. She had regular contractions and spontaneous rupture of membranes. She tested positive for Covid-19 with PCR, but had no symptoms. She had developed gestational diabetes in this pregnancy. Her BMI was 27.08 kg/m^2^. Her medication included insulin, iron, and prenatal vitamins. She had a normal delivery and was discharged from the hospital the following day with no postpartum complications.

Two days later (postpartum day two), she presented in the emergency ward due to dyspnea. She had developed upper respiratory tract symptoms the same day she was discharged from the hospital and had increased difficulty with breathing. At the time of admission, she was hypoxemic (SaO_2_ 88% on 3 L O_2_*→*93% on 6 L O_2_ on oxymask), tachypneic (RR 42*→*45 breaths/minute), and normotensive (BP 130/71 mmHg) and had a normal HR 95 beats/minute with no fever. The laboratory samples indicated mild leukocytosis and CRP 107 mg/L. The ABG showed respiratory alkalosis. Treatment with antibiotics, LMWH, fluids, and a first dose of chloroquine phosphate was administered before she was transported to an IMC unit.

Within three hours, her clinical condition deteriorated with increased RR 50 breaths/minute, SaO_2_ 91% on 10 L O_2_, and fever 38.2°C. The CT scan demonstrated general multifocal pneumonic infiltrate and situs inversus but no pulmonary embolism. She was admitted to the ICU, and endotracheal intubation was made due to moderate ARDS. She was then transported to Karolinska University Hospital for further treatment. The dose of LMWH was increased, chloroquine phosphate was discontinued, and prone ventilation was initiated. On HD 8 (ICU day 6), her clinical condition was stable and extubation from ETI was performed, but due to immediate decompensation, she was immediately reintubated. Although the blood cultures did not show any specific growth of pathogens, the CRP levels continued to be high >100 mg/L and broad-spectrum antibiotics were therefore given.

On HD 11 (ICU day 9), she received a tracheostomy, and on HD 14 (ICU day 12), step-down from the ICU to IMC was done. On HD 17, she was decannulated from the tracheostomy and transported to a medical ward for further rehabilitation. At HD 21, she was discharged from the hospital. At the time of writing, the patient has fully recovered from her respiratory insufficiency although fatigue remains as a symptom.

### 2.5. Case 4

A 36-year-old, gravida 2, para 1, with Middle Eastern background, presented at the obstetrical ward, in gestational week 40 + 6. She was in a latent phase with regular spontaneous contractions. At the time of admission, she was subfebrile and the cardiotocography indicated fetal tachycardia. She was normotensive (110/70 mmHg), tachycardic (HR 110 beats/minute), and tachypneic (RR 26); the saturation level was not documented. Three days before admission, she had the onset of fever, arthralgia, and upper respiratory tract symptoms. She tested positive with PCR for Covid-19. She had no previous medical history besides migraine, and her BMI was 27.27 kg/m^2^. Her only medication was prenatal vitamins and iron substitution.

She was first observed for a couple of hours with regular monitoring and was given intravenous paracetamol, terbutaline, and morphine to release the pain from the contractions during the latent phase. As there was no progress in her labor, the decision for induction with a Foley catheter was taken, and three hours later, there was a spontaneous rupture of membranes with meconium. As there was a clinical suspicion of chorioamnionitis, treatment with intravenous antibiotics was initiated. The progress of labor was slow, and the CTG had an abnormal pattern with continuous tachycardia and episodes with decreased variability, although the fetal scalp blood test indicated normal lactate levels, but as the patient developed fever (38.5°C), the decision for a cesarean section after 20 h from the beginning of induction was made. The operation was uncomplicated, although meconium had contaminated the amniotic fluid; fortunately, the neonate was doing fine with an Apgar of 9 : 10 : 10.

For the first three postpartum days, she was continuously subfebrile, tachycardic, and hypotensive, but with additional fluid, antibiotics, and LMWH, her clinical condition was stable. On HD 6, she suddenly deteriorated in her respiration; her RR increased (23 breath/minute) and SaO_2_ decreased (89% on air). She received 6 L O_2_ on oxymask, but due to augmented dyspnea, fever, CRP 234 mg/L, ABG indicating respiratory alkalosis, and an increased demand of oxygen (SaO_2_ 89% on 6 L O_2_*→*SaO_2_ 96% on 10 L O_2_), she was transferred to an IMC for further monitoring. A CT scan was made and demonstrated general multifocal pneumonic infiltrates but no pulmonary embolism.

On the following day, HD 7, she developed diarrhea, and the respiratory rate increased to 40-45 breaths/minute. She was transferred to the ICU, and on HD 8, her respiratory insufficiency was defined as moderate ARDS, and ETI was made to secure her ventilation. The echocardiography did not indicate any signs of right or left ventricular failure. The prone position was made for better ventilation.

Her clinical condition gradually improved, and on HD 15 (ICU day 9), she was successfully extubated, and on HD 16, she was transferred to a medical ward for further rehabilitation and treatment. On HD 19, she was discharged from the hospital. At the time of writing, she has fully recovered from her respiratory insufficiency.

### 2.6. Case 5

A 30-year-old, gravida 4, para 2, with Asian background presented at a general hospital in gestational week 22 + 3. In her obstetrical history, she had one early spontaneous miscarriage, one intrauterine fetal death (IUFD), and one full term delivery. Her medical history related to the current pregnancy included a known protein S deficiency, diet-treated gestational diabetes, and a BMI of 33.75 kg/m^2^. Her only medication was iron substitution. For the previous three days, she had developed symptoms including cough, fever, and dyspnea which gradually had worsened. Due to deterioration of her respiration with increased dyspnea, she decided to contact the hospital.

At the time of admission, she was dyspneic, severely hypoxemic (70% SaO_2_ on air*→*80% on 15 L O_2_ oxymask), tachypneic (50-55 breaths/minute), tachycardic (120 beats/minute), and normotensive (120/65 mmHg). She had fever 39.4°C, increased CRP, and hypokalemia, and the ABG indicated acute uncompensated respiratory acidosis. Within an hour, she was intubated and transported to Karolinska University Hospital for further treatment at the ICU. Before the transport, the first dose of prenatal betamethasone and intravenous antibiotics was given. The PCR test for Covid-19 was positive.

Once at the ICU, an echocardiography was made with no signs of right or left ventricular failure. The chest imaging demonstrated typical general multifocal pneumonic infiltrates but no pulmonary embolism or edema. High doses with LMWH were initiated. There was an ethical dilemma on whether the pregnancy should be continued or the fetus should be aborted on maternal indication; a multidisciplinary conference was therefore held for discussion. The joint decision was to continue the pregnancy and give the second dose of antenatal betamethasone.

On HD 5 (ICU day 5), the patient's CRP levels increased (>200 mg/L) and so did the oxygen demand with episodes of hypercapnia and a low pO_2_/FiO_2_ ratio, indicating severe ARDS. The intravenous antibiotics were broadened even though no pathogens were found in the cultures. The LMWH was increased to high doses due to low antifactor Xa concentration, and the supine-prone position was needed to optimize the ventilation. At HD 13 (ICU day 13), her clinical condition had improved, and a tracheostomy was performed.

On ICU day 15, she once again deteriorated, and extubation from the ETI could not be performed. In the culture from the tracheostomy, growth of Klebsiella aerogenes was found, and additional antibiotic and antifungal treatments were therefore given. The LMWH doses were increased due to continuously low antifactor Xa levels. On ICU day 18, the mechanical ventilation support was not enough to substitute for her pulmonary insufficiency, indicating the initiation of venovenus extracorporeal membrane oxygenation (v-v ECMO).

On ECMO day 5 (HD 22), she was considered to have recovered from Covid-19 due to four negative PCR test in a row, and her pulmonary condition was now assessed to be affected by a “normal” bacterial pneumonia. Over the following ten days, her clinical condition improved, and on ECMO day 14 (HD31), she was decannulated from ECMO. At HD 35, in gestational week 27 + 2, she was transferred from the ECMO ward to the ICU for further treatment.

Five hours later she suddenly developed seizures and lost consciousness. A bolus dose of magnesium infusion and diazepam were given with the aim to stop the seizures with little improvement. She did not respond to pain stimulation, and an eye deviation to the right was observed. Urgent preparations for a CT scan and an electroencephalogram (EEG) were made. Just before the transfer, the telemetry signaled arrhythmia with irregular heart rhythm and acute bradycardia, with a loss of pulse. The blood pressure decreased and so did the saturation, resulting in cardiac arrest. Cardiopulmonary resuscitation was initiated and preparation for perimortem caesarean section at the ICU was made. However, she had a return of spontaneous circulation (ROSC) and thereby could be transported to the operation theater. A healthy preterm neonate was delivered in gestational week 27 + 3 and was transferred to the neonatal intensive care unit.

After the cesarean section, she was replaced in v-v ECMO (replaced-ECMO day 1) and was in need of several blood and plasma transfusions due to hemodynamic instability. Heparin infusion was therefore temporarily stopped. On HD 38, an exploratory laparotomy was made as a damage control due to continuous need of transfusion and suspicion of intra-abdominal bleeding. The main bleeding came from rectus abdominis muscle tears. Hemostasis was achieved by sutures and diathermy, and the abdominal cavity was packed with several abdominal packs. This was not enough though to maintain hemodynamic stability, and there have been three more reoperations to try to control diffuse bleeding from the abdominal muscles and peritoneum. An angiography during the last relook on HD 43 was made, indicating extravasation from an internal iliac arterial genital branch, and in addition, diffuse bleeding from omental and subcutaneous tissue as from the rectus muscles was still seen. The hemodynamic instability was addressed to severe “clinical” coagulopathy although activated partial thromboplastin time (APTT), rotational thromboelastometry (ROTEM), and fibrinogen levels were normal. Continuous renal replacement therapy (CRRT) was initiated to provide renal support due to acute kidney injury.

The bleeding eventually subsided, and her clinical condition gradually improved; on HD 52 (replaced-ECMO day 17), she was successfully decannulated from ECMO and transferred to the ICU for further treatment. On HD 56, decannulation of the tracheostomy was performed, the CRRT was discontinued, and the patient was transferred to a regular medical ward. At the time of writing, her clinical condition is now stable although she is still hospitalized with ongoing rehabilitation.

## 3. Discussion

Our aim with presenting these five cases of pregnant or newly delivered women with Covid-19 is to add more knowledge of the clinical outcomes that pregnant women with severe and critical Covid-19 disease may develop. In comparison to other case series and reports that have been published on the subject, Covid-19 and pregnancy, we can also see that there are several similarities in the patient groups when it comes to the terms of clinical presentation, risk factors, and disease progression.

In a meta-analysis by Zaigham and Andersson on 108 pregnant women with symptomatic Covid-19, it was found that the mean age for the women was 29-32 years, the main symptom was fever (59%), and elevated CRP above 10 mg/L was seen in more than 70% [6]. This is in line with our cases (Table 1).

When it comes to imaging diagnostics, Yan et al. found that 96% (104) of their pregnant patients had abnormal findings on the CT scan, described as patchy shadowing or ground glass opacity [4]. In our cases, all five went through a CT scan and atypical multifocal pneumonic infiltrates with patchy shadowing were found. Characteristic infiltrates such as ground glass opacities have also been described in the nonpregnant population [13], and it could therefore be advisable to prefer CT scans instead of conventional X-rays in pregnant women, both for better diagnostics of Covid-19 and to exclude pulmonary embolism.

The role of the echocardiogram in pregnant women with Covid-19 can also be discussed. Arentz et al. published a case series on 21 nonpregnant patients that required intensive care due to Covid-19, and they found that approximately 33% of the patients developed cardiomyopathy with echocardiographic signs of globally reduced left ventricular systolic function [14]. Juusela et al. presented a case series on two critically ill pregnant women with cardiomyopathy, and as a conclusion, they suggest that echocardiogram in pregnant women should be recommended, especially in those who required intensive care [15]. In our five cases, echocardiogram was performed in all of them, and no heart failure or cardiomyopathy was found.

Diabetes, hypertension, and high BMI have been announced as risk factors for developing a more severe and critical Covid-19 disease in the nonpregnant population [12, 16]. Pregnant women, with underlying health conditions, such as diabetes, hypertensive disorders, and high BMI, have been considered vulnerable to Covid-19 [17–19]. In this case series, three out of five patients had gestational diabetes and all five had BMI > 25. For comparison, according to the Swedish Pregnancy Register, 3.1% of the pregnant women with BMI between 25.0 and 29.9 had gestational diabetes and 2.8% of the total pregnant population had gestational diabetes; meanwhile, 27% were overweight with BMI > 25, in Sweden 2018 [20].

This report is based on a case series with limited material; in order to make any significant conclusions, more detailed information on symptoms, treatment, and outcomes for pregnant and postpartum women managed in intensive care is needed.

## Figures and Tables

**Table 1:** Summary.

Age (years)	29	36	30	36	30
Race/ethnicity	Middle Eastern	African	Asian	Middle Eastern	Asian
BMI (kg/m^2^)	25.6	29.69	27.08	27.27	33.75
Obstetrical history	G1, P0	G5, P2	G2, P1	G2, P1	G4, P2
Gestational week at time of admission	21 + 4	22 + 5	37 + 4	40 + 6	22 + 3
Chief complaint	Fever, nausea, diarrhea, cough	Cough, fatigue, arthralgia, myalgia, nausea, vomits	Dyspnea, upper respiratory tract symptoms	Fever, dyspnea	Dyspnea, cough, fever
Medical comorbidities	None	(i) Gestational diabetes (ii) Gestational hypothyroidism	Gestational diabetes Situs inversus	None	(i) Gestational diabetes (ii) Protein-S deficiency
Notable admission laboratory results	CRP 37 mg/L K+ 3.3mmol/L pH 7.48 pCO_2_ 3.8 kPa pO_2_ 8.2 kPa	CRP 44 mg/L K+ 2.8 mmol/L pH 7.33 pCO_2_ 3.3 kPa BE -12 mmol/L	CRP 107 mg/L pH 7.14 pCO_2_ 10.4 kPa pO_2_ 8.8 kPa BE -4 mmol/L	CRP 50 mg/L pH 7.39 pCO_2_ 3.7 kPa PO_2_ 14.9 kPa BE -8 mmol/L	CRP 103 mg/L K+ 3.0 mmol/L pH 7.26 pCO_2_ 4.2 kPa pO_2_ 5.1 kPa BE -2 mmol/L
Number of days from onset of symptoms to intubation	No intubation, NHF 12 L/min	13	1	8	6
Number of hospital days	15	22	21	19	Still hospitalized
Adjunctive therapy	(i) LMWH (ii) Cefotaxime (iii) Fluid resuscitation	(i) LMWH (ii) Cefotaxime (iii) Meropenem (iv) Fluid resuscitation	(i) LMWH (ii) Cefotaxime (iii) Chloroquine phosphate (iv) Linezolid (v) Fluid resuscitation	(i) LMWH (ii) Piperacillin/tazobactam (iii) Meropenem (iv) Clindamycin (v) Fluid resuscitation	(i) LMWH (ii) Heparin infusion (iii) Cefotaxime (iv) Piperacillin/tazobactam (v) Meropenem (vi) Fluid resuscitation (vii) Dialysis (viii) ECMO
Antenatal steroids	Not given	Not given	Not given	Not given	Betamethasone
Additional clinical examinations	CT scan (multifocal pneumonic infiltrates) ECO (normal)	CT scan (multifocal pneumonic infiltrates) ECO (hyperdynamic left ventricle)	CT scan (multifocal pneumonic infiltrates) ECO (normal)	CT scan (multifocal pneumonic infiltrates) ECO (normal)	CT scan (multifocal pneumonic infiltrates) ECO (normal)
